# A phase 1 first‐in‐human study of GS‐0189, an anti‐signal regulatory protein alpha (SIRPα) monoclonal antibody, in patients with relapsed/refractory (R/R) non‐Hodgkin lymphoma (NHL)

**DOI:** 10.1002/jha2.687

**Published:** 2023-04-07

**Authors:** Mayur Narkhede, Nancy L. Bartlett, Sami Ibrahimi, Leslie Popplewell, Anna Seto, Jamie Bates, Yeonju Lee, Vaishnavi Ganti, Ling Han, Tianling Chen, Manish R. Patel

**Affiliations:** ^1^ Division of Hematology/Oncology Department of Medicine University of Alabama at Birmingham Birmingham Alabama USA; ^2^ Department of Medicine Division of Oncology Washington University School of Medicine St. Louis Missouri USA; ^3^ Stephenson Cancer Center University of Oklahoma Health Sciences Center Oklahoma City Oklahoma USA; ^4^ Department of Hematology City of Hope National Medical Center Duarte California USA; ^5^ Clinical Development Gilead Sciences, Inc Foster City California USA; ^6^ Research, Gilead Sciences, Inc Foster City California USA; ^7^ Biomarker Sciences Gilead Sciences, Inc Foster City California USA; ^8^ Clinical Pharmacology Gilead Sciences, Inc Foster City California USA; ^9^ Biostatistics Gilead Sciences, Inc Foster City California USA; ^10^ Department of Drug Development Florida Cancer Specialists/Sarah Cannon Research Institute Sarasota Florida USA

**Keywords:** CD47, GS‐0189, SIRPα, non‐Hodgkin lymphoma, monoclonal antibodies

## Abstract

Signal regulatory protein alpha (SIRPα) is the receptor for cluster of differentiation (CD)47, a potent “don't eat me” signal for macrophages. Disruption of CD47‐SIRPα signaling in the presence of prophagocytic signals can lead to enhanced phagocytosis of tumor cells, resulting in a direct antitumor effect; agents targeting this pathway have shown efficacy in non‐Hodgkin lymphoma (NHL) and other tumor types. GS‐0189 is a novel anti‐SIRPα humanized monoclonal antibody. Here we report: (1) clinical safety, preliminary activity, and pharmacokinetics of GS‐0189 as monotherapy and in combination with rituximab from a phase 1 clinical trial in patients with relapsed/refractory NHL (NCT04502706, SRP001); (2) in vitro characterization of GS‐0189 binding to SIRPα; and (3) in vitro phagocytic activity. Clinically, GS‐0189 was well tolerated in patients with relapsed/refractory NHL with evidence of clinical activity in combination with rituximab. Receptor occupancy (RO) of GS‐0189 was highly variable in NHL patients; binding affinity studies showed significantly higher affinity for SIRPα variant 1 than variant 2, consistent with RO in patient and healthy donor samples. In vitro phagocytosis induced by GS‐0189 was also SIRPα variant–dependent. Although clinical development of GS‐0189 was discontinued, the CD47‐SIRPα signaling pathway remains a promising therapeutic target and should continue to be explored.

## 1 INTRODUCTION

Non‐Hodgkin lymphoma (NHL) is one of the most common cancers in the United States, accounting for about 4% of all cancers [1]. Systemic treatment options include chemotherapy, immuno‐ and targeted therapy, chimeric antigen receptor (CAR) T‐cell therapy, and stem cell transplant [2]. Many patients have relapsed/refractory (R/R) disease following frontline treatment for NHL [3, 4, 5, 6]. The overall prognosis and long‐term survival for R/R patients after multiple lines of therapy are often poor [7, 8, 9]. CAR T‐cell therapy provides impressive response rates in heavily pretreated R/R follicular and other B‐cell NHLs, but toxicities, complicated logistics, limited access, and delays in delivering treatment may limit use of this therapy in many patients [10, 11, 12]. The development of more effective and tolerable therapies for R/R NHL represents a high unmet medical need.

Signal regulatory protein alpha (SIRPα) is a receptor expressed on phagocytic cells—such as macrophages, neutrophils, monocytes, and dendritic cells—that binds to the antiphagocytic signal cluster of differentiation (CD)47 [13, 14], initiating a signalling cascade within the phagocyte that inhibits engulfment, preventing destruction of “self” by the innate immune system [15]. Various tumor types overexpress CD47 [16, 17, 18], enabling evasion of macrophage‐mediated phagocytosis [17], making the SIRPα‐CD47 interaction an attractive therapeutic target [19]. Increased pro‐phagocytic signals induced by other agents may greatly enhance activity of SIRP‐CD47 blockade, providing strong support for combination therapy [19].

Human SIRPα is highly polymorphic in the immunoglobulin (Ig) V‐like domain [20], the ligand‐binding domain for CD47 [14, 21]. In humans, the SIRPα protein is found as two major variants, V1 and V2, presenting as homozygotes (SIRPα^V1/V1^ and SIRPα^V2/V2^) or heterozygotes (SIRPα^V1/V2^) [22]. About 42%–47% of the population is heterozygous, with proportions of SIRPα^V1/V1^ and SIRPα^V2/V2^ being more variable across populations [22].

GS‐0189 is a novel humanized IgG1 monoclonal anti‐SIRPα antibody with an aglycosylated (inert) Fc region that blocks recognition by Fcγ receptors and prevents the phagocytosis of SIRPα‐expressing cells. GS‐0189 was designed as an alternative to magrolimab, an anti‐CD47 monoclonal antibody (mAb) in clinical development in hematologic malignancies and solid tumors, as it had been found not to impact red blood cells in preclinical experiments [23]. Here we report: (1) clinical safety, preliminary activity, and pharmacokinetics (PK) of GS‐0189 as monotherapy and combination therapy with rituximab, from a phase 1 first‐in‐human (FIH) clinical trial in patients with R/R NHL (NCT04502706); (2) in vitro characterization of GS‐0189 binding potency to SIRPα variants; (3) and in vitro phagocytic activity of macrophages derived from donors possessing different SIRPα variants relative to the activity of a control pan‐SIRP blocking antibody with an inert Fc, KWAR23.

## 2 METHODS

### 2.1 Clinical study

#### 2.1.1 Design and participants

This was an open‐label FIH trial to evaluate GS‐0189 safety, PK, and preliminary efficacy of monotherapy and in combination with rituximab in patients with select R/R NHL histologies (Supplemental Methods; Figure S1): here, we report monotherapy dose escalation (MDE) and dose escalation in combination with rituximab (CDE). Eligible patients were ≥18 years of age with diffuse large B‐cell, follicular, mantle cell, or marginal zone lymphomas R/R to ≥2 prior lines of therapy. Patients with prior autologous hematopoietic cell transplantation and/or CAR T‐cell therapy were eligible. All patients provided written informed consent prior to participation in the study. The study was conducted in accordance with the protocol and with US Food and Drug Administration Guidelines, International Conference on Harmonisation Good Clinical Practice guidelines, the Declaration of Helsinki, and all local health authority and Institutional Review Board/Independent Ethics Committee requirements for each study site.

#### 2.1.2 Procedures

GS‐0189 was administered intravenously every 2 weeks. Planned treatment in MDE cohorts was GS‐0189 at 10, 30, or 100 mg. Planned treatment in CDE cohorts was GS‐0189 100 to 3000 mg in combination with rituximab 375 mg/m^2^ on days 1, 8, 15, and 22 of cycle 1; day 1 of cycles 2 to 5; and day 1 of every other cycle thereafter. Dose‐limiting toxicity (DLT) was assessed during the DLT observation period (cycle 1, days 1–28). GS‐0189 serum concentrations were measured using an electrochemiluminescent assay (lower limit of quantification, 30 ng/mL) developed and validated by QPS LLC (Newark, DE). See Supplemental Methods for details. Serum GS‐0189 concentration‐time data were analyzed by a noncompartmental approach using Phoenix WinNonlin (Version 8.2; Certara, Princeton, NJ). Blood samples for GS‐0189 immunogenicity were collected and analyzed (see Supplemental Methods). Rituximab PK were not determined.

#### 2.1.3 Endpoints

The primary endpoint was incidence of adverse events defined by National Cancer Institute Common Terminology Criteria for Adverse Events, Version 5.0. Secondary endpoints included GS‐0189 PK, objective response rate per Lugano response criteria [24], duration of response (DOR), and progression‐free survival (PFS).

### 2.2 Statistical analysis

Statistical analyses were performed on all patients who received ≥1 dose of GS‐0189. For categorical variables, frequencies and percentages were calculated.

### 2.3 Nonclinical and ex vivo assays

Antibodies and reagents used in these studies are listed in Table S1.

#### 2.3.1 Receptor occupancy assay

The receptor occupancy (RO) was evaluated in blood from both healthy donors and NHL patients. SIRPα RO was assessed using a free receptor format with allophycocyanin conjugated anti‐human CD172ab antibody. The mean fluorescence intensity values for each fluorochrome in the panel (CD45, CD14, CD15, 7AAD, and CD172ab) were measured.

Blood from NHL patients in MDE cohorts were collected for RO evaluation. Clinical RO data were acquired using an FACSCanto flow cytometer. Ultra‐rainbow beads were collected with the same instrument setting as the samples to generate molecules of equivalent fluorochrome.

#### 2.3.2 GS‐0189 and KWAR23 binding to SIRPα variants

Binding experiments quantifying the affinity of GS‐0189 or KWAR23 for recombinant SIRPα variants were performed on either a Biacore T100 or T200 instrument using a CM5 sensor chip. GS‐0189 and KWAR23 as active control [25, 26] were captured and regenerated according to manufacturer instructions.

SIRPα^V1^ and SIRPα^V2^ were injected using maximum concentrations of either 0.3 μM and three‐fold serial dilutions for the higher affinity interactions, or 3 μM and 4‐fold serial dilutions for the lower affinity interactions. Data were fitted to a simple kinetic model to derive *k_on_*, *k_off_*, and K_D_, using the relationship K_D_ = *k_off_* / *k_on_*.

#### 2.3.3 In vitro phagocytosis assay

In vitro phagocytosis was evaluated using peripheral blood mononuclear cells (PBMCs) from healthy donors expressing SIRPα^V1/V1^, SIRPα^V1/V2^, or SIRPα^V2/V2^ variants (determined by Sanger sequencing) that were differentiated into macrophages by treating with macrophage colony‐stimulating factor for 7 days. Macrophages were then co‐cultured with Raji Burkitt's lymphoma cells or DLD‐1 colorectal adenocarcinoma cells that had been labeled with CellTrace carboxyfluorescein succinimidyl ester (CFSE) to achieve an effector:target ratio of 1:2. After 2 h of incubation, with anti‐SIRP or isotype control antibodies, phagocytosis was quantified as the percentage of CD11b^+^ macrophages that were positive for CFSE. Phagocytic index was calculated as fold‐increase relative to vehicle control.

#### 2.3.4 SIRPα genotyping assay

NHL patients (SRP001) and healthy donor PBMC samples were used for genotyping assay by Sanger sequencing. Six different Sanger sequencing reactions were performed using the primers (Table S2) to generate conclusive Sanger sequencing data.

DNA was sequenced using the 3730 Genetic Analyzer. SnapGene Viewer was used to review the chromatograms, and Clustal Omega to compare the DNA and translated RNA sequences. See Supplemental Methods for details.

## 3 RESULTS

### 3.1 Clinical study results

Nine patients were enrolled and treated between December 2, 2020 and March 31, 2022, with six patients enrolled in MDE cohorts (10 mg GS‐0189 [*n* = 1], MDE1; 30 mg GS‐0189 [*n* = 1], MDE2; and 100 mg GS‐0189 [*n* = 4] MDE3) and 3 in the first CDE cohort (100 mg GS‐0189 + rituximab, CDE1). After the first CDE cohort, the decision was made to terminate the SRP001 study (see Discussion for rationale). Patient characteristics are shown in Table 1. Reasons for GS‐0189 discontinuation were patient's decision (*n* = 2) and progressive disease (*n* = 7). Reasons for study discontinuation were death (*n* = 1), withdrawn consent (*n* = 2), and study terminated by sponsor (*n* = 6). Treatment‐emergent AEs (TEAEs) occurred in all but one patient across treatment groups. The most common TEAEs occurring in >1 patient in any group were infusion‐related reactions (IRRs), anemia, and neutropenia. Of the 4grade 3 TEAEs (pain, fatigue, anemia, and neutropenia), only neutropenia was related to GS‐0189. There were no DLTs, grade 4 TEAEs, or events leading to death (Table 2). One patient in MDE3 had grade 3 anemia on day 3 that resolved on day 4 after 2 units of packed red blood cells and was deemed unrelated to GS‐0189. Another patient in MDE3 with baseline grade 1 anemia had grade 2 anemia deemed related to GS‐0189 and did not require intervention. IRRs occurred in 6 patients (4 patients with IRRs related to GS‐0189, 1 with an IRR to rituximab, and 1 with IRRs to both drugs); all IRRs were grade 1 except 1 patient with symptom of grade 2 chills (Table 3). Four patients had grade 3 or 4 treatment‐emergent hematologic laboratory abnormalities, including decreased hemoglobin, lymphocytes, neutrophils, and leukocytes in 1 patient; and transient lymphocytes decrease in all 3 patients in CDE1 within 24 h of infusion (1 with grade 2 lymphocyte decrease at screening), which resolved within cycle 1 (Table S3).

**TABLE 1:** Demographics and baseline disease characteristics.

Patient	GS‐0189 dose**^1^**	Age	Sex	NHL type	Prior anti‐CD20 Ab / rituximab refractory	SIRPα variant	Number of prior lines of anti‐cancer therapy	Time from end of last therapy to study treatment start (months)
10001	10 mg	65	F	DLBCL	Yes / No	V2/V2	3	20.6
10002	30 mg	46	M	FL	Yes / No	V1/V2	3	3.1
10003	100 mg	70	M	DLBCL	Yes / Yes	V1/V1	3	1.1
10004	100 mg	58	F	FL	Yes / Yes	V2/V2	4	1.3
10005	100 mg	66	M	FL	Yes / No	V1/V1	5	56.1
10006	100 mg	59	M	FL	Yes / No	V1/V1	2	32.2
10007	100 mg + Rituximab	51	F	FL	Yes / No	V1/V1	2	7.2
10008	100 mg + Rituximab	47	M	DLBCL	Yes / No	V1/V2	3	3.9
10009	100 mg + Rituximab	71	M	FL	Yes / No	V1/V2	3	7.2

^1^ Administered IV. Rituximab dose, 375 mg/m^2^ IV.

Abbreviations: Ab, antibody; CD, cluster of differentiation; DLBCL, diffuse large B‐cell lymphoma; F, female; FL, follicular lymphoma; IV, intravenously; M, male; NHL, non‐Hodgkin lymphoma; SIRPα, signal regulatory protein alpha; V, variant.

**TABLE 2:** Treatment‐emergent adverse events.^1^

	MDE				CDE
*n* (%)	GS‐0189 10 mg, MDE1 (*N* = 1)	GS‐0189 30 mg, MDE2 (*N* = 1)	GS‐0189 100 mg, MDE3 (*N* = 4)	MDE Total (*N* = 6)	GS‐0189 100 mg + rituximab, CDE1 (*N* = 3)
Any TEAE	1 (100.0)	1 (100.0)	3 (75.0)	5 (83.3)	3 (100.0)
Serious TEAE	0	0	1 (25.0)	1 (16.7)	0
Treatment‐related TEAE	1 (100.0)	0	3 (75.0)	4 (66.7)	2 (66.7)
Grade 1 or 2	1 (100.0)	0	2 (50.0)	3 (50.0)	2 (66.7)
Grade 3 or 4^2^	0	0	1 (25.0)	1 (16.7)	0
TEAE leading to dose interruption of GS‐0189	0	0	0	0	1 (33.0)^3^
TEAE leading to death	0	0	0	0	0
Dose‐limiting toxicity	0	0	0	0	0
TEAEs occurring in > 1 patient in any group					
Infusion‐related reaction	1 (100.0)	0	2 (50.0)	3 (50.0)	3 (100.0)
Anemia	0	0	2 (50.0)	2 (33.3)	0
Neutropenia	0	0	2 (50.0)	2 (33.3)	0

^1^ TEAEs are AEs with onset dates on or after the first dose of study drug and up to 30 days after study drug discontinuation.

^2^ One anemia and 1 neutropenia TEAE were grade 3. There were no grade 4 TEAEs.

^3^ Dose interruption occurred twice in 1 patient due to COVID‐19 infection and an automobile accident.

Abbreviations: CDE, combination dose escalation; MDE, monotherapy dose escalation; TEAE, treatment‐emergent adverse event.

**TABLE 3:** Infusion‐related reactions in the SRP001 study.

	MDE				CDE
*n* (%)	GS‐018910 mg, MDE1 (*N* = 1)	GS‐018930 mg, MDE2 (*N* = 1)	GS‐0189100 mg, MDE3 (*N* = 4)	MDE Total (*N* = 6)	GS‐0189 100 mg + rituximab, CDE1 (*N* = 3)
Any infusion‐related reaction^1^	1 (100.0)	0	2 (50.0)	3 (50.0)	3 (100.0)
Chills	0	0	2 (50.0)	2 (33.3)	3 (100.0)^1^
Pruritus	1 (100.0)	0	0	1 (16.7)	1 (33.3)
Back pain	0	0	1 (25.0)	1 (16.7)	0
Dizziness	0	0	0	0	1 (33.3)
Feeling cold	0	0	1 (25.0)	1 (16.7)	0
Nausea	1 (100.0)	0	0	1 (16.7)	0
Neck pain	0	0	0	0	1 (33.3)
Night sweats	0	0	0	0	1 (33.3)
Vomiting	1 (100.0)	0	0	1 (16.7)	0

^1^ One infusion‐related reaction was grade 2 (chills, in CDE1); all other infusion‐related reaction symptoms were grade 1.

Abbreviations: CDE, combination dose escalation; MDE, monotherapy dose escalation.

One patient with stage 4 follicular lymphoma (FL) in CDE1 who had previously relapsed after 2 lines of rituximab‐containing regimens achieved a complete response (CR) on study day 82 with a DOR of 5.19 months and PFS of 7.85 months. The DOR and PFS were censored at the time of last adequate tumor assessment. Study treatment was discontinued due to patient decision, which coincided with the time of sponsor decision to discontinue the study. Two patients had stable disease (1 each, from MDE2 and CDE1) with estimated PFS of 5.55 and 3.71 months, respectively; 5 had progressive disease (Table 4); and 1 withdrew consent prior to the first response assessment.

**TABLE 4:** Overall response in the SRP001 study.

	MDE			CDE
Parameter, n (%)	GS‐0189 10 mg (*N* = 1)	GS‐0189 30 mg (*N* = 1)	GS‐0189 100 mg (*N* = 4)^1^	GS‐0189 100 mg + rituximab (*N* = 3)
Overall response rate	0	0	0	1 (33.3)
Best overall response				
Complete response	0	0	0	1 (33.3)
Partial response	0	0	0	0
Stable disease	0	1 (100.0)	0	1 (33.3)
Progressive disease	1 (100.0)	0	3 (75.0)	1 (33.3)

^1^ One patient withdrew consent prior to first response assessment. Abbreviations: CDE, combination dose escalation; MDE, monotherapy dose escalation.

### 3.2 Pharmacokinetics

**FIGURE 1:**
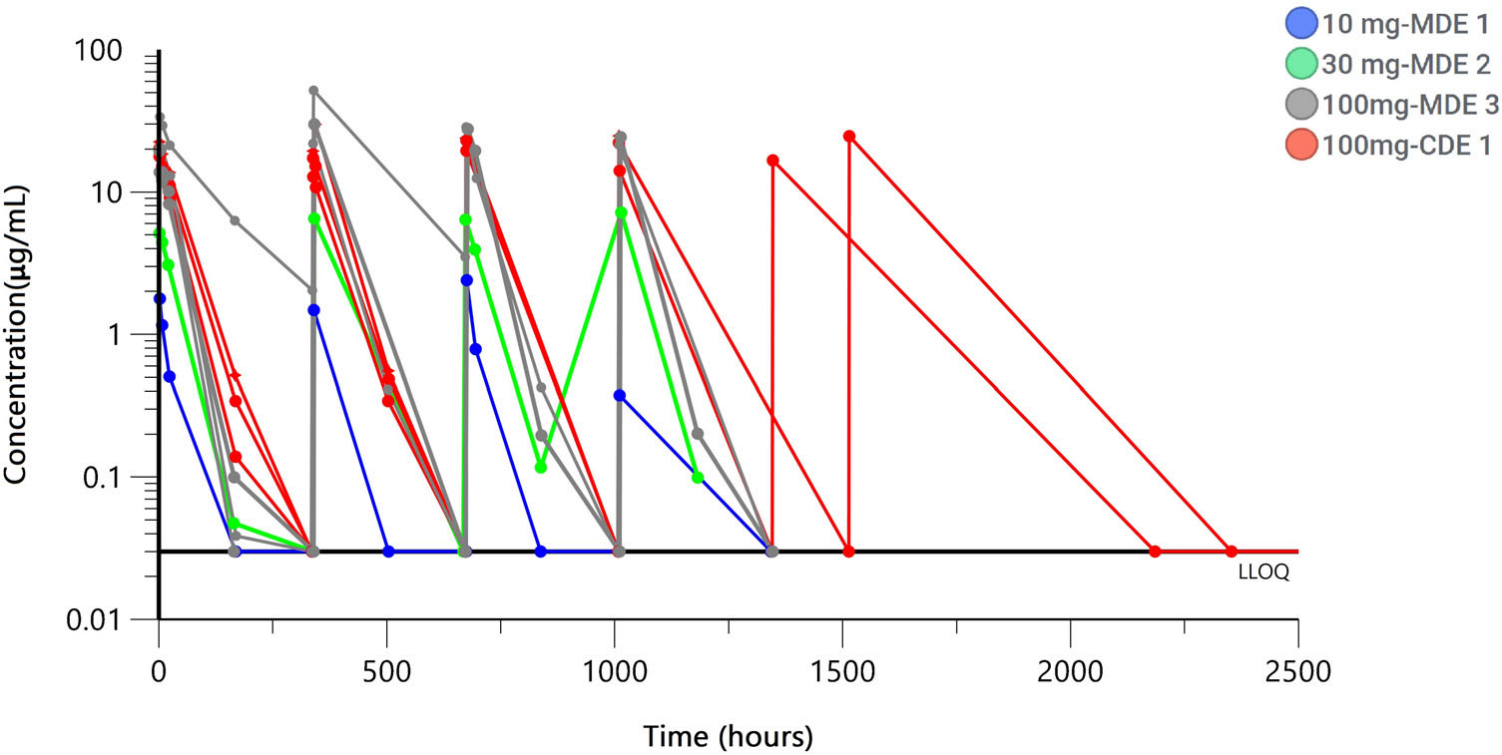
Concentration‐time profiles for GS‐0189 across dose groups 10 to 100 mg. Abbreviation: CDE, combination dose escalation; LLOQ, lower limit of quantification; MDE, monotherapy dose escalation.

Concentration‐time profiles for patients in MDE1, 2, and 3 and CDE1 are shown in Figure 1. Relevant PK parameters for MDE and CDE (Cohort 1) are listed in Table 5. The area under the curves for GS‐0189 serum concentration‐time profiles in the given dose range of 10 to 100 mg were lower than projected based on cyno PK‐PD studies. This could potentially be due to target‐mediated drug disposition for the antibody in humans. GS‐0189 RO over time from three patients in CDE1 is shown in Figure S2 for comparison with concentration‐time profile. The antidrug antibody incidence rate for GS‐0189 cannot be interpreted due to a small sample size.

**TABLE 5:** Median pharmacokinetic parameters for GS‐0189 post first dose (cycle 1, day 1).

Dose (mg)	*N*	AUC_last_ (μg/mL*hour)	C_max_ (μg/mL)	V_ss_ (L)
10	1	21.7	1.78	NC
30	1	184	5.14	5.61
100	4	615	20.1	3.92
100	3	768	20.2	5.43

Abbreviations: AUC_last_, area under the curve from the time of dosing to the time of the last measurable (positive) concentration; C_max_, maximum concentration; V_ss_, apparent volume of distribution at equilibrium after intravenous administration.

### 3.3 Differential binding of GS‐0189 in SIRPα variants

**FIGURE 2:**
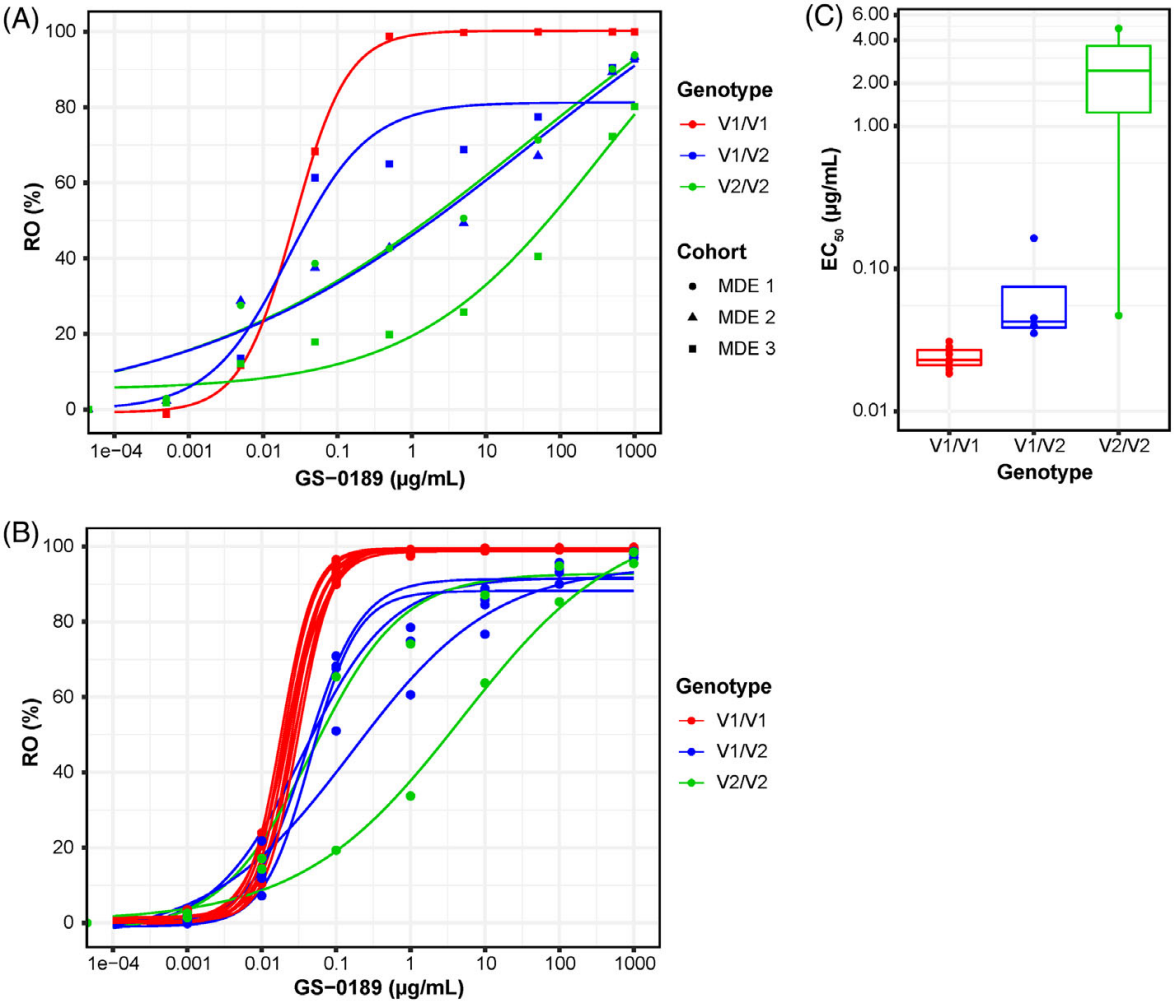
GS‐0189 binding profiles of NHL patients from study SRP001 and healthy donors with different SIRPα allelic variants. (A) GS‐0189 RO titration curves with identified SIRPα genotypes from MDE1, MDE2, and MDE3 cohorts. (B) GS‐0189 binding profiles on CD14^+^ monocytes from 15 commercial healthy donors. (C) calculated GS‐0189 concentration at which 50% of SIRPα receptors on CD14^+^ monocytes are occupied in healthy donors, grouped by SIRPα allelic variants: EC_50_ (μg/mL) of SIRPα^V1/V1^ = 0.024 ± 0.004; SIRPα^V1/V2^ = 0.071 ± 0.062; SIRPα^V2/V2^ = 2.44 ± 3.39. Abbreviations: CD, cluster of differentiation; EC_50_, half‐maximal effective concentration; MDE, monotherapy dose escalation; NHL, non‐Hodgkin lymphoma; RO, receptor occupancy; SIRPα, signal regulatory protein alpha; V, variant.

RO of GS‐0189 showed highly variable binding of GS‐0189 across samples from 5 NHL patients (Figure 2A). Sanger sequencing of SIRPα variants from PBMCs of healthy donors (*n* = 15) and patients (*n* = 9; Table 1 for patient results) revealed three allelic variants: homozygous SIRPα^V1/V1^ (*n* = 9 donors, *n* = 3 patients), homozygous SIRPα^V2/V2^ (*n* = 2 donors, *n* = 2 patients), and heterozygous SIRPα^V1/V2^ (*n* = 4 donors, *n* = 4 patients). RO binding curves from healthy donors showed GS‐0189 bound to the homozygous SIRPα^V1/V1^ variant 3‐ and 102‐fold more strongly than to SIRPα^V1/V2^ and SIRPα^V2/V2^ variants, respectively (Figures 2B,C), and was consistent with binding profiles obtained from patients (Figure 2A).

#### 3.3.1 GS‐0189 and KWAR23 binding to SIRPα variants

**FIGURE 3:**
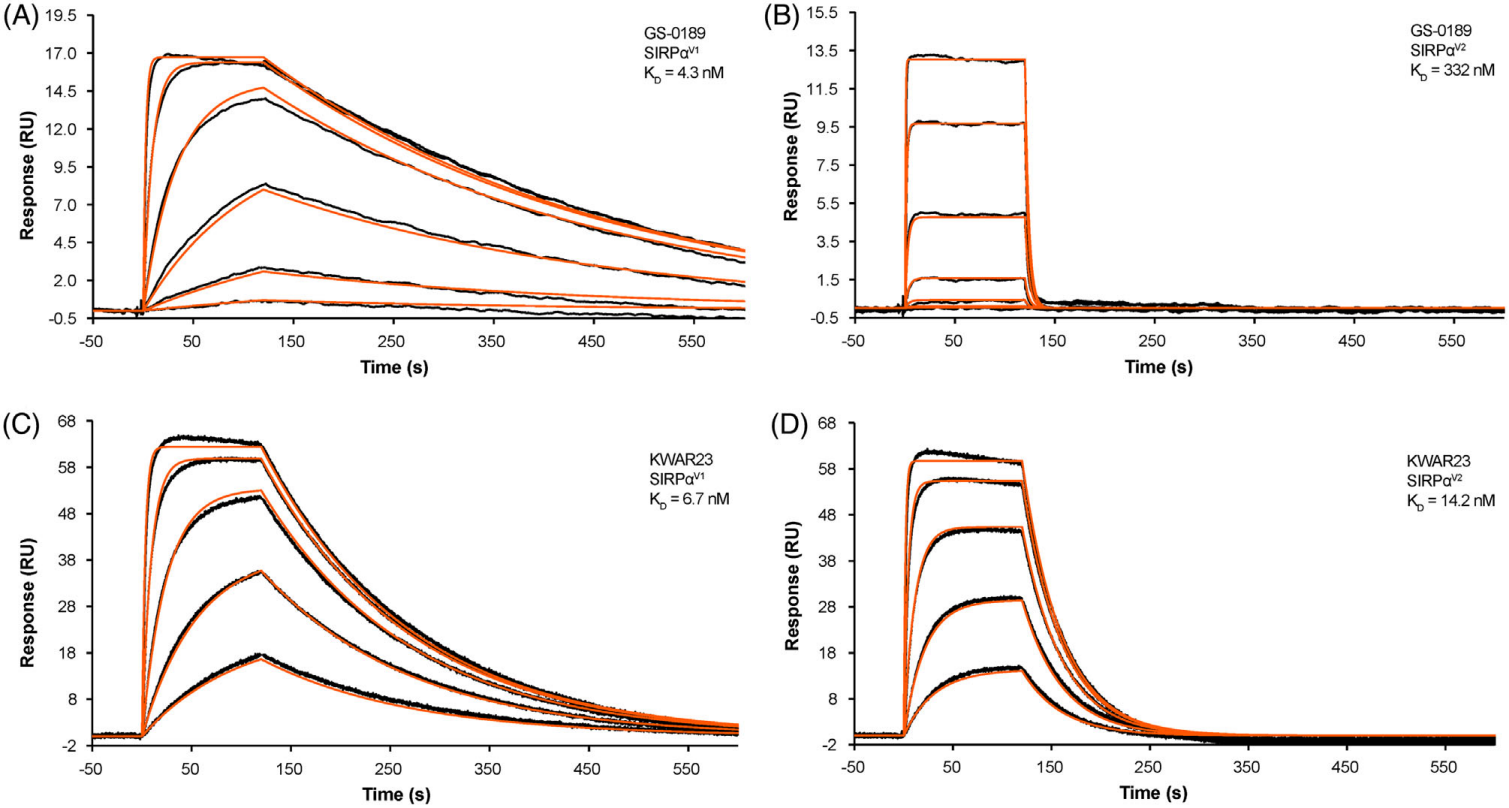
GS‐0189 binding to SIRP isoforms and variants shown by sensogram for GS‐0189 (A and B) or KWAR23 (C and D) binding to SIRPα^V1^ (A and C), and SIRPα^V2^ (B and D). The highest concentration injected was 3 μM for lower affinity interactions, and 0.3 μM for higher affinity interactions for SIRPα^V1^ and SIRPα^V2^ for GS‐0189. The highest concentration injected was 0.3 μM for all SIRP variants for KWAR23. Black lines denote binding data; orange lines represent the kinetic fit. Abbreviations: SIRPα, signal regulatory protein alpha.

Binding affinities of GS‐0189 to recombinant SIRPα^V1^ and SIRPα^V2^ compared with those of KWAR23 are shown by kinetic and equilibrium constants (Table 6) and sensograms (Figure 3). GS‐0189 had a 77‐fold higher affinity for SIRPα^V1^ (K_D_ = 4.3 nM) than for SIRPα^V2^ (K_D_ = 332 nM). KWAR23 demonstrated a narrower range of affinities to SIRPα variants: K_D_ = 6.7 nM for SIRPα^V1^ and K_D_ = 14.2 nM for SIRPα^V2^.

**TABLE 6:** Thermodynamic and kinetic parameters.

Antibody	Antigen	*k_on_* ^1^(M^−1^ s^−1^)	*k_off_* ^1^ (s^−1^)	K_D_ ^1^, ^2^ (nM)
GS‐0189	SIRPα^V1^	6.2E + 05	2.6E‐03	4.3
	SIRPα^V2^	6.5E + 05	2.5E‐01	332
KWAR23	SIRPα^V1^	1.0E + 06	6.7E‐03	6.7
	SIRPα^V2^	1.8E + 06	2.5E‐02	14.2

^1^ Average of 2‐3 independent experiments. For all interactions studied, values for *k_on_*,*k_off_*, and K_D_ varied less than 2‐fold between experiments.

^2^ The equilibrium dissociation constant K_D_ = *k_off_*/*k_on_*.

#### 3.3.2 Potency of GS‐0189 in phagocytosis assays is dependent on SIRPα polymorphism

**FIGURE 4:**
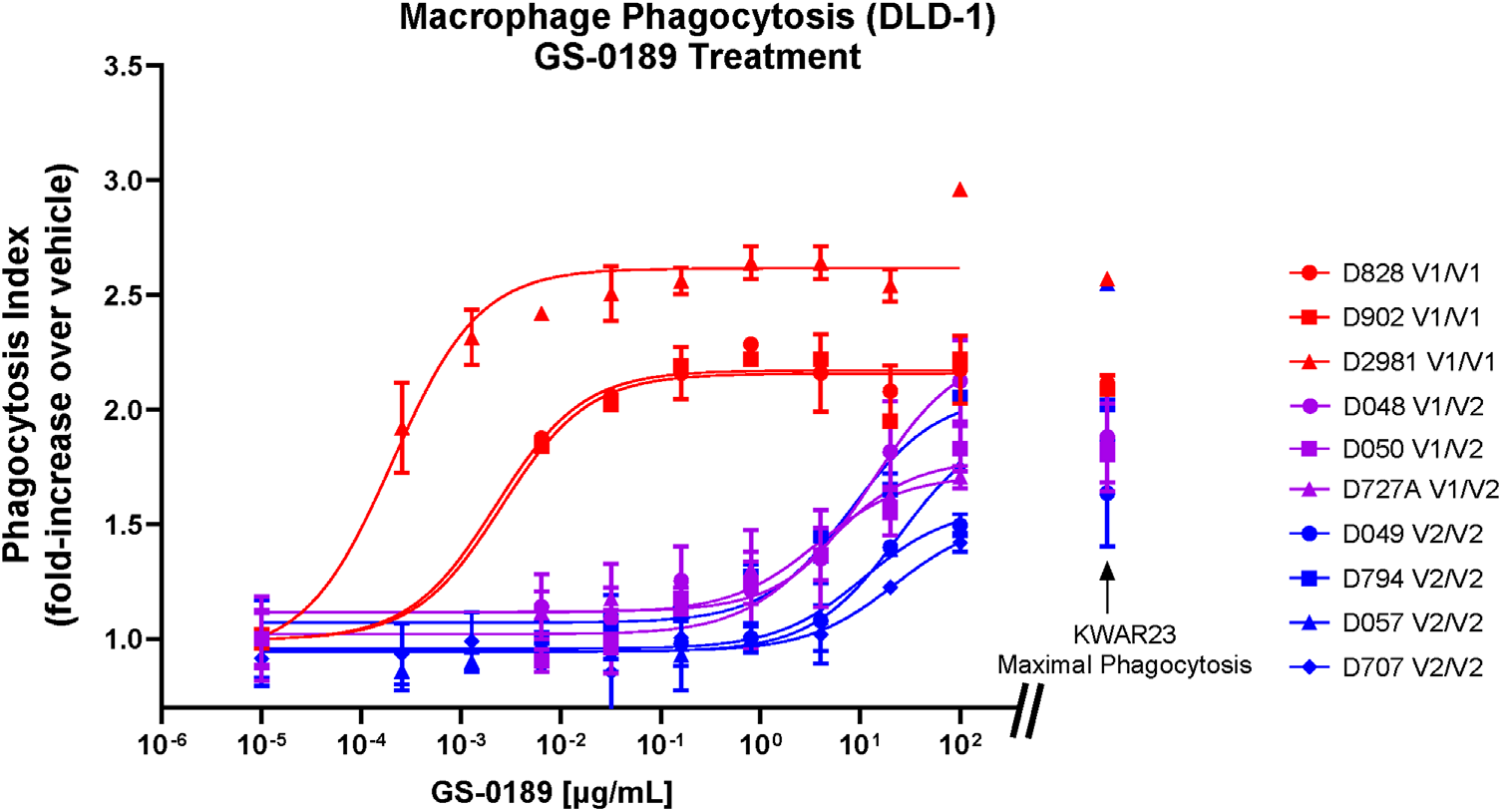
Phagocytosis induced by anti‐SIRP. CFSE‐labeled DLD‐1 cells were co‐cultured with macrophages isolated from healthy donor PBMCs that were homozygous or heterozygous for SIRPα^V1^ or SIRPα^V2^ as described in the legend. GS‐0189 dose‐dependently induced phagocytosis. For additional controls, separate co‐cultures were treated with an Fc‐inert pan anti‐SIRPα antibody (KWAR23) at maximally efficacious concentrations. After a 2‐hour co‐culture, cells were analyzed by flow cytometry to determine frequency of CD11b^+^CFSE^+^ cells. The frequency of macrophages positive for CFSE was normalized to vehicle to generate the phagocytic index. Samples were tested in duplicate and presented as mean ± SD. Abbreviations: CD, cluster of differentiation; CFSE, carboxyfluorescein succinimidyl ester; PBMC, peripheral blood mononuclear cell; SD, standard deviation; V, variant.

Maximal phagocytosis of Raji cells by macrophages from PBMC donors with SIRPα^V1/V1^ and SIRPα^V2/V2^ variants was induced by GS‐0189 combined with rituximab (Figure S3), with only a small increase in maximal phagocytosis induced by the combination versus rituximab alone. Therefore, we identified a human colorectal cancer cell line DLD‐1 for which phagocytosis was more dependent on inhibition of the CD47‐SIRPα axis and for which phagocytosis was significantly induced in response to CD47‐SIRPα blockade alone [25]. The ability of GS‐0189 to potentiate phagocytosis of DLD‐1 cells by human PBMC‐derived macrophages from donors with different SIRPα variants was dose dependent (Figure 4). The average half‐maximal effective concentration (EC_50_) of GS‐0189 with macrophages from donors expressing SIRPα^V1/V1^ was 0.02 μg/mL, expressing SIRPα^V1/V2^ was 13.8 μg/mL, and expressing SIRPα^V2/V2^ was 9.5 μg/mL. KWAR23 induced phagocytosis across SIRPα variants with EC_50_ ranging from 0.04 to 0.10 μg/mL (Figure S4). Regardless of SIRPα genotype, maximal phagocytosis induced by GS‐0189 was equivalent to that induced by KWAR23 at concentrations above 10 μg/mL (Figure 4).

## 4 DISCUSSION

GS‐0189 up to 100 mg as monotherapy and in combination with rituximab was well tolerated by patients with R/R NHL in this phase 1 study. There were no DLTs, no grade 4 TEAEs, and no deaths related to TEAEs. Since SIRPα expression is mainly on macrophages and GS‐0189 does not have a functional Fc, it was theorized that GS‐0189 would result in less anemia compared to most CD47‐targeting agents [23, 27]. In SRP001, anemia occurred in 2 patients, 1 of whom had anemia at baseline. Lymphocyte count decreases observed with GS‐0189 in combination with rituximab were transient and resolved quickly. Although lymphopenia has been reported with rituximab monotherapy [28], the causality of this phenomenon will remain unclear unless a randomized clinical study is conducted to evaluate each drug's contribution.

GS‐0189 is an anti‐SIRPα antibody with an aglycosylated (inert) Fc region that was theorized to benefit from the presence of another drug that provides an “eat me” signal, such as rituximab, to induce phagocytic activity. In this clinical study, there was no observed response in patients enrolled in the MDE cohorts. In CDE1, one patient achieved a CR at approximately 12 weeks, which was maintained until study termination. This patient had stage 4 FL, relapsed on 2 prior lines of rituximab‐containing regimens, and was homozygous for the SIRPα^V1^ variant. This could suggest that there may still be therapeutic benefit by adding a SIRPα‐blocking agent to rituximab in patients who have relapsed on prior rituximab therapy. However, since this is an observation for a single patient, further clinical evaluation in patients with NHL may be needed to interrogate the mechanism of SIRPα‐blocking agents in combination with rituximab.

Affinity of GS‐0189 for the SIRPα^V1^ variant was nearly 2 orders of magnitude higher than for SIRPα^V2^, which was evident in the highly variable RO of GS‐0189 to SIRPα in samples from study patients, prompting further exploration using healthy donor samples. Genotyping for SIRPα variants in healthy donors coupled with binding affinity and RO data confirmed that GS‐0189 binding was weaker to SIRPα^V2^ than SIRPα^V1^; and the heterogeneity in the study patient RO findings could be explained by the genotyping of study patients. Concordant with SIRPα‐binding affinities, induction of phagocytosis by GS‐0189 as a single agent differed by more than 2 orders of magnitude between macrophages from donors expressing SIRPα^V1/V1^ and those expressing SIRPα^V1/V2^ or SIRPα^V2/V2^ (EC_50_ <0.1 μg/mL vs. ∼ >10 μg/mL, respectively). KWAR23 induced phagocytosis of the DLD‐1 human colorectal cancer cell line across SIRPα variants with equivalent potencies, suggesting that there is no inherent difference in phagocytic capacity of macrophages from patients with different SIRPα variants. Comparable ability of GS‐0189 combined with cetuximab to potentiate phagocytosis by PBMC‐derived macrophages from SIRPα^V1/V1^‐ and SIRPα^V2/V2^‐expressing donors was previously demonstrated using HT‐29 cells, the epidermal growth factor receptor–expressing human tumor cell line, as the target cell [23]. Consistent with this observation, we found that maximal phagocytosis of Raji cells induced by GS‐0189 in combination with rituximab was similar across PBMC donor genotypes. However, the difference between maximal phagocytosis induced by rituximab alone versus the combination was small, leaving little room to evaluate the contribution of GS‐0189. Systematic functional assessment of GS‐0189 monotherapy by dose confirmed the difference in phagocytic index of GS‐0189 by SIRPα variant. These data suggest that higher GS‐0189 doses might have been necessary to achieve concentrations needed for phagocytic activity in patients harboring SIRPα^V1/V2^ and SIRPα^V2/V2^ genotypes.

GS‐0189 was intended as a low anemia‐risk alternative to magrolimab. With priming‐dose regimen and clinical management, acute anemia observed with magrolimab is no longer expected to limit magrolimab clinical development [29, 30]. Therefore, the decision was made to terminate the SRP001 study and discontinue clinical development of GS‐0189. Nevertheless, the CD47‐SIRPα interaction/phagocytic mechanism remains a promising target for treatment of patients with solid tumors and hematologic malignancies and should continue to be explored.

## AUTHOR CONTRIBUTIONS

MN, NLB, SI, LP, AS, JB, VG, TC, and MP conceived of or designed the study. MN, LP, JB, YL, and MP acquired and provided data.

All authors analyzed or interpreted the data, drafted, or critically reviewed the manuscript, approved the final version, and agreed to be accountable for all aspects of the work.

## CONFLICT OF INTEREST STATEMENT

Mayur Narkhede reports institutional research funding from EUSA Therapeutics, Genmab, and Roche‐Genentech; and attending advisory boards and honoraria from ADC Therapeutics, T.G. Therapeutics, and Seagen. Nancy L. Bartlett reports research funding from ADC Therapeutics, Affimed, Bristol Meyers Squibb, Celgene, Forty‐Seven, Gilead Sciences, Inc., Immune Design, Janssen, Kite Pharma, Merck, Millennium, Pfizer, Pharmacyclics, Roche‐Genentech, and Seattle Genetics. Sami Ibrahimi reports stock in Karyopharm Therapeutics. Leslie Popplewell reports no conflict of interest. Anna Seto, Jamie Bates, Yeonju Lee, Vaishnavi Ganti, Ling Han, and Tianling Chen all report employment with and stock in Gilead Sciences, Inc. Manish Patel reports leadership at ION Pharma; honoraria from Pfizer, Pharmacyclics, Bayer, Janssen Oncology, Genentech, and Adaptive Biotechnologies; consulting or advisory roles for Pharmacyclics/Janssen and Pfizer/EMD Serono; attending speakers’ bureaus for Exelixis, Roche‐Genentech, Taiho Pharmaceutical, and Celgene; and research funding from Acerta Pharma, ADC Therapeutics, Agenus, Aileron Therapeutics, AstraZeneca, BioNTech AG, Boehringer Ingelheim, Celgene, Checkpoint Therapeutics, CicloMed, Clovis Oncology, Cyteir Therapeutics, Daiichi Sankyo, Lilly, EMD Serono, Evelo Therapeutics, FORMA Therapeutics, Roche‐Genentech, Gilead Sciences, Inc., GlaxoSmithKline, H3 Biomedicine, Hengrui Therapeutics, Hutchison MediPharma, Ignyta, Incyte, Jacobio, Janssen, Klus Pharma, Kymab, Loxo, LSK Biopartners, Lycera, Macrogenics, Merck, Millennium, Mirati Therapeutics, Moderna Therapeutics, Pfizer, Placon, Portola Pharmaceuticals, Prelude Therapeutics, Ribon Therapeutics, Seven and Eight Biopharmaceuticals, Syndax, Taiho Pharmaceutical, Takeda, Tesaro, TopAlliance Biosciences Inc., Vigeo, ORIC, Artios, Treadwell, Mabspace, IgM Biosciences, Puretech, BioTheryX, Black Diamond Therapeutics, NGM Biopharmaceuticals, Novartis, Nurix, Relay Therapeutics, Samumed, Silicon Therapeutics, TeneoBio, Zymeworks, Olema, Adagene, Astellas, NGM, Accutar Biotech, TeneoBio, Compugen, Immunogen, and Blueprint Pharmaceuticals.

## Supporting information

Supporting Information

## Data Availability

Gilead Sciences shares anonymized individual patient data upon request or as required by law or regulation with qualified external researchers based on submitted curriculum vitae and reflecting non conflict of interest. The request proposal must also include a statistician. Approval of such requests is at Gilead Science's discretion and is dependent on the nature of the request, the merit of the research proposed, the availability of the data, and the intended use of the data. Data requests should be sent to datarequest@gilead.com.
