# Supplementary material for: A phase 1 first‐in‐human study of GS‐0189, an anti‐signal regulatory protein alpha (SIRPα) monoclonal antibody, in patients with relapsed/refractory (R/R) non‐Hodgkin lymphoma (NHL)
Source: EJHaem. 2023 Apr 7;4(2):370–80. doi: 10.1002/jha2.687 (PMC10188468; doi:10.1002/jha2.687)

**SUPPLEMENTAL APPENDIX—Table of Contents**

**Supplemental Methods**

**SUPPLEMENTAL TABLE 1** List of antibodies and reagents used in this manuscript

**SUPPLEMENTAL TABLE 2** List of primers used for SIRPα genotyping using sanger sequencing

**SUPPLEMENTAL TABLE 3** Grade 3 or 4 laboratory abnormalities

**SUPPLEMENTAL FIGURE 1** Study Design Schema

**SUPPLEMENTAL FIGURE 2** Receptor occupancy results from the CDE1 cohort during treatment

**SUPPLEMENTAL FIGURE 3** Phagocytosis of Raji cells induced by anti-SIRP and anti-CD20 antibodies

**SUPPLEMENTAL FIGURE 4** Phagocytosis of DLD-1 cells induced by KWAR23

**SUPPLEMENTAL METHODS**

**Clinical Trial SRP001**

*Study design and patients*

This was an open-label, first-in-human dose escalation trial consisting of monotherapy dose escalation (MDE), combination dose escalation (CDE), pharmacokinetics (PK) evaluation, alternative schedule evaluation (ASE), and diffuse large B-cell lymphoma (DLBCL) expansion. Eligible patients were ≥ 18 years of age who had DLBCL, follicular lymphoma, mantle cell lymphoma, or marginal zone lymphoma with measurable disease (Lugano criteria [Cheson 2014]) relapsed/refractory (R/R) to at least ≥ 2 prior lines of therapy, Eastern Cooperative Oncology Group performance score of 0 to 2, and adequate renal and hepatic function; patients with indolent lymphomas were required to be candidates for systemic treatment. Prior autologous hematopoietic cell transplantation and/or chimeric antigen receptor T-cell therapy (> 3 months prior to initial study treatment) and patients with transformed lymphomas were permitted.

*Procedures*

In the MDE part, patients were treated with GS-0189 monotherapy doses of 10, 30, or 100 mg every 2 weeks. In the CDE part, patients were treated with GS-0189 from 100 to 3000 mg in combination with rituximab 375 mg/m^2^. In the PK evaluation part, patients were treated with doses < 300 mg GS-0189 monotherapy (cycle 0 day 1) followed by GS-0189 at the highest dose previously deemed safe by the Clinical Trial Steering Committee (CTSC) in the CDE part in combination with rituximab 375 mg/m^2^ (cycle 1 day 1). In the ASE part, patients were treated with GS-0189 (as determined by the totality of safety, PK, and pharmacodynamics [PD] data from preceding cohorts) every 4 weeks in combination with rituximab 375 mg/m^2^. In the DLBCL expansion part, recommended dose and schedule for GS-0189 was determined based on an analysis of the totality of safety, PK, and PD data from the preceding cohorts, and was administered in combination with rituximab 375 mg/m^2^. The ASE and DLBCL expansion parts would not be initiated until dose escalation was complete, and the PK evaluation part would not be initiated until the second CDE dose level was deemed safe by the CTSC. Both GS-0189 and rituximab were administered intravenously. Dose-limiting toxicity (DLT) was assessed during the DLT observation period (cycle 1 day 1 to cycle 1 day 28), and was defined as any grade 3 or greater adverse event (AE) that occurs during the 4-week DLT observation period regardless of attribution, including those that worsened in severity from pretreatment baseline with the exception of the nonhaematologic and haematologic criteria:

- Grade 3 or 4 electrolyte abnormalities that resolve to grade ≤ 2 with supportive care within 72 hours and are not associated with other clinically significant consequences. However, abnormalities that persist longer than 72 hours should be considered a DLT.
- Grade 3 elevation in bilirubin, alanine aminotransferase, aspartate aminotransferase, or alkaline phosphatase or electrolyte abnormalities that resolve to grade ≤ 2 with supportive care within 1 week and are not associated with other clinically significant consequences.
- Transient grade 3 nausea, vomiting, diarrhea, local reactions, influenza-like symptoms, myalgias, fever, headache, or acute pain that resolves to grade ≤ 2 within 72 hours with supportive care.
- Grade 3 fatigue that resolves to grade ≤ 2 within 2 weeks.
- Grade 3 infusion-related reaction that resolves to grade ≤ 1 within 72 hours.
- Grade 3 or 4 lymphopenia.
- Grade 3 or 4 neutropenia that resolves to grade ≤ 2 within 5 days without growth factors.
- Grade 3 thrombocytopenia in the absence of clinically significant bleeding. Note that grade 4 thrombocytopenia or grade 3 thrombocytopenia with grade ≥ 2 bleeding is a DLT.
- Grade 3 anaemia; however, grade 3 haemolytic anaemia that is medically significant, requires hospitalisation or prolongation of existing hospitalisation, is disabling, or limits self-care activities of daily life is considered a DLT.

Study treatment was continued until disease progression, unacceptable drug toxicity, or patient/physician decision to discontinue. AEs that occurred after the first dose of study drug through 30 days after the last dose of study drug were assessed according to National Cancer Institute Common Terminology Criteria for Adverse Events Version 5.0. Screening bone marrow aspirate/biopsy was performed in patients with known bone marrow disease, and postbaseline bone marrow biopsy for confirmation of complete response and/or at disease progression was required only if a biopsy had not been completed in the prior 4 weeks. Responses were evaluated using Lugano response criteria. (Cheson 2014)

*Outcomes*

The primary endpoints were safety and tolerability as measured by AEs. Secondary endpoints included GS-0189 PK; immunogenicity; efficacy measures including objective response rate (ORR), duration of response (DOR), progression-free survival (PFS), time to progression (TTP), and overall survival (OS); and definition of a recommended dose for GS-0189 in combination with rituximab in patients with R/R non-Hodgkin lymphoma (NHL). Exploratory endpoints were immune cell efficacy and tumor penetration, preliminary efficacy in molecular subtypes of NHL, and the impact of patient signal regulatory protein alpha (SIRPα) genotype on receptor occupancy (RO) and response.

*Statistical analysis*

Statistical analyses were performed on all patients who received ≥ 1 dose of GS-0189. The DLT analysis was conducted in DLT-evaluable patients, which included any patient who either (i) experienced a DLT at any time after initiation of the first infusion of GS-0189, (ii) completed at least 2 infusions of GS-0189 in the MDE cohort, (iii) completed at least 2 infusions of GS-0189 and at least 2 infusions of rituximab in the CDE cohort, or (iv) completed 1 infusion of GS-0189 and at least 2 infusions of rituximab in the ASE cohorts. Safety was assessed through summaries of death, DLTs, AEs, changes in laboratory test results, and exposure to GS-0189. The number of patients initially planned for inclusion in this study was up to 81 patients, as follows: MDE, 6 patients; CDE, up to 30 patients for the 5 GS-0189 dose levels; PK evaluation, up to 18 patients; ASE, up to 12 patients; DLBCL expansion, up to 15 patients. All analyses were conducted by treatment (GS-0189 monotherapy and GS-0189 + rituximab), unless otherwise noted.

For continuous variables, the mean, standard deviation, median, Q1, Q3, and ranges were determined. For categorical variables, the frequency and percentage in each category were calculated.

Endpoints included in the efficacy analysis were ORR, PFS, OS, and TTP, and analyses were conducted on all enrolled patients who received at least 1 dose of the study drug. The analysis of DOR was conducted on patients who achieved an objective response (defined as complete response + partial response determined by Lugano response criteria [Cheson 2014]).

**Pharmacokinetic, antidrug antibody methods and analysis**

Blood samples for the determination of serum GS-0189 concentrations were collected at pre-dose, end of infusion, and 6, 24, 72, 168 (day 8), and 336 (day 15) hours after first infusion of cycle 1. On cycle 1 day 15, 1- and 6-hour post-dose samples were collected. Cycle 2 pre- and end-of-infusion samples were collected on days 1 and 15. Cycle 3 and beyond, pre- and end-of-infusion samples were collected. A bioanalytical method was developed and validated by QPS LLC (Newark, DE) for the determination of GS-0189 concentrations in human serum, which met the expectations presented in the 2018 FDA guidance for bioanalytical method validation. The method consisted of an electrochemiluminescence (ECL) immunoassay. In this assay, analyte-capturing antibody, a biotinylated anti-idiotypic antibody that binds to an epitope site of GS-0189, was attached to the microtiter plates pre-coated with streptavidin. Samples (including standards and quality controls) were diluted with a minimum required dilution of 5, then added and captured on the plate. A secondary analyte detection antibody, a ruthenium-conjugated anti-idiotypic antibody that binds to a different epitope site of GS-0189, was then added and incubated on the plate. After washing to remove unbound analyte detection antibody, the wells of the plate were subjected to an electric field; the resulting chemiluminescence was measured in relative light units (RLU), and the signal was proportional to the quantity of GS-0189 present in the sample. GS-0189 concentrations were determined by interpolation on a calibration curve with a calibration range of 30 to 900 ng/mL. The calibration curve equation was fitted to the calibration results using four-parameter Marquardt regression with a weighting factor of 1/y², where y is ECL signal. All samples were analysed within the established storage stability. Serum GS-0189 concentration-time data were analysed by a noncompartmental approach using Phoenix® WinNonlin® (Version 8.2; Certara, Princeton, NJ). PK of rituximab were not evaluated.

**SIRPα genotyping assay**

Sanger sequencing of 15 peripheral blood mononuclear cell (PBMC) samples from healthy donors (AllCells, Alameda, CA) and PBMCs from 6 patients (enrolled in MDE1, MDE2, and MDE3 cohorts), and buccal swabs from 3 patients (enrolled in the CDE1 cohort), were performed. PCR amplification of the target region was carried out using polymerase chain reaction primers pairs 5’-CTACATTGGATATTCTTGG and 5’- CAGTTGGTGTTTGGTGATG, and the following reaction conditions were applied: (i) preheating at 95°C for 5 min; (ii) amplification consisting of 3 cycles at 95°C for 30 seconds, 55°C for 30 seconds, and 72°C for 1 min, then 32 cycles at 95°C for 30 sec, 50°C for 30 sec, and 50°C for 30 sec; (iii) an extension at 72°C for 1 min; and (iv) a final extension at 72°C for 10 min.

### GS-0189 and KWAR23 Binding to SIRPα variants

Binding experiments quantifying the affinity of GS-0189 or KWAR23 to recombinant SIRPα variants were performed on either a Biacore T100 or T200 instrument using a CM5 sensor chip. Human antibody capture kit was used to immobilise anti-human antibody on these surfaces with standard amine coupling chemistry. GS-0189 and KWAR23 as active control (Ring 2017; Voets 2019) were captured and regenerated according to manufacturer instructions at densities ranging from 35–170 RU and 115–130 RU, respectively.

For all experiments, the instrument was first stabilised by multiple blank buffer injections before injection of analyte samples SIRPα variant 1 (SIRPα^V1^) and SIRPα^V2^. SIRPα^V1^ and SIRPα^V2^ were injected using maximum concentrations of either 0.3 μM and 3-fold serial dilutions for the higher affinity interactions, or 3 μM and 4-fold serial dilutions for the lower affinity interactions. After each injection, surfaces were regenerated with 3 M MgCl_2_.

Experiments were performed at 25°C with running buffer composed of 10 mM HEPES pH 7.4, 150 mM NaCl, 0.005% P20, and 0.1 mg/mL bovine serum albumin. Samples were injected for a contact time of 120 seconds. Dissociation was then monitored for 600 seconds. Data were fitted to a simple kinetic model to derive *k_on_*, *k_off_*, and K_D_, using the relationship K_D_ = *k_off /_ k_on_*

**In vitro phagocytosis assay**

In vitro phagocytosis was evaluated using healthy donor PBMCs. Monocytes were isolated from PBMCs expressing SIRPα^V1/V1^, SIRPα^V1/V2^, or SIRPα^V2/V2^ variants (determined by Sanger sequencing), differentiated to macrophages by incubating 2 × 10^6^ cells per well in a 6-well low attachment plate in 50 ng/mL of human recombinant macrophage colony-stimulating factor for 7 days. Macrophages were harvested using TrypLE, washed, counted, plated on low-attachment 96-well plates at 2 × 10^4^ cells per well, and rested 1 hour prior to co-culture with tumor cells. Raji Burkitt’s lymphoma cells or DLD-1 colorectal adenocarcinoma cells were cultured in complete RPMI-1640 or DMEM media, respectively, harvested using TrypLE detachment media, and labeled with 0.5 µg/mL of CellTrace carboxyfluorescein succinimidyl ester (CFSE) in phosphate-buffered saline (PBS) at 37°C for 30 minutes. Labeled cells were quenched by incubating with complete DMEM media and incubated at 37°C for an additional 30 minutes, and were added to resting macrophages to achieve an effector:target ratio of 1:2. Anti-SIRPα or isotype antibody was added, with a maximum concentration of 100 µg/mL. After 2 hours of incubation, supernatants were removed by centrifuging for 5 minutes at 400 × g and resuspending in 100 µL of PBS with 1:2000 fixable viability dye, and cells were incubated at room temperature for 10 minutes. Cells were washed and resuspended in 100 µL fluorescence-activated cell sorter (FACS) buffer containing mouse Fc-block and incubated on ice for 10 minutes. Anti-mouse CD11b APC was added to each well and incubated on ice in the dark for 30 minutes. Cells were washed and resuspended in 200 µL of FACS buffer and acquired on a BD Fortessa flow cytometer. Phagocytosis was quantified as the percentage of CD11b^+^ macrophages that were positive for CFSE. Phagocytic index was calculated as fold-increase relative to vehicle control.

**RO assay**

Whole blood from healthy donors (AllCells, LLC) was collected in K_2_EDTA tubes at room temperature and placed immediately on ice for processing. GS-0189 was added in whole blood at concentrations from 1000 to 0 µg/mL, and then incubated with Fc Block (Human TruStain, BioLegend Cat #: 422302) for 10–12 minutes on ice. SIRPα RO was performed using a free receptor format with anti-human CD172ab (SIRPα/β) antibody (clone SE5A5, BioLegend Cat #: 323810). Antibodies were added to the whole blood: anti-human CD45 V510 (leukocyte common antigen; Clone HI30, BioLegend Cat #304036), anti-human CD14 V421 (LPS receptor; Clone M5E2, BioLegend Cat # 301830), anti-human CD15 FITC (Clone HI98, BioLegend Cat #: 301904), 7AAD (viability stain; BioLegend, Cat # 430404), and anti-human CD172ab APC. Samples were washed twice with FACS buffer and resuspended in FACS buffer for acquisition. RO was calculated as follows:

$$RO\left( \% \right)=\left( 1-\frac{{MFI of free SIRP\alpha}_{test}-{MFI of background}_{test}}{{MFI of free SIRP\alpha}_{baseline}-{MFI of background}_{baseline}} \right)\times100$$

Clinical RO data were acquired using a FACSCanto flow cytometer. Ultra-rainbow beads (Spherotech, Catalog number: URCP-38-2K) were collected with the same instrument setting as the samples to generate molecules of equivalent fluorochrome. RO was calculated as follows:

$$RO(\%)=\left( 1-\frac{M{OEF of free SIRP\alpha}_{test}-{MOEF of background}_{test}}{{MOEF of free SIRP\alpha}_{baseline}-{MOEF of background}_{baseline}} \right)\times100$$

**SUPPLEMENTAL TABLE 1** Antibodies, reagents, instruments, and data analysis software used in this manuscript

| **Method** | **Name** | **Clone**  **(antibodies)** | **Source** | **Cat #** |
| --- | --- | --- | --- | --- |
| RO assay | Whole blood from healthy donors |  | AllCells, LLC |  |
|  | Human TruStain FcX™ (Fc Receptor Blocking Solution) |  | BioLegend | 422302 |
|  | Anti-human CD172ab (SIRPα/β) APC | SE5A5 | BioLegend | 323810 |
|  | Anti-human CD45 V510 | HI30 | BioLegend | 304036 |
|  | Anti-human CD14 V421 | M5E2 | BioLegend | 301830 |
|  | Anti-human CD15 FITC | HI98 | BioLegend | 301904 |
|  | 7AAD |  | BioLegend | 420404 |
|  | Ultra-rainbow beads |  | Spherotech | URCP- 38-2K |
| SPR and phagocytosis assays | Anti-human SIRPα, Fc inert | KWAR23 | Creative Biolabs | TAB-453CT |
|  | Human antibody capture kit |  | Cytiva | BR100839 |
|  | Human recombinant M-CSF |  | RnD | 216MC100CF |
|  | Raji Burkitt’s lymphoma cells |  | ATCC | ATCC-CCL-86 |
|  | DLD-1 |  | ATCC | ATCC-CCL-221 |
|  | RPMI-1640 media |  | Gibco | 11875119 |
|  | DMEM media |  | Gibco | 11965118 |
|  | CellTrace CFSE |  | ThermoFisher | C34570 |
|  | Zombie UV™ Fixable Viability Kit |  | BioLegend | 423108 |
|  | FACS buffer |  | BD | 554656 |
|  | Purified rat anti-mouse CD16/CD32 (Mouse BD Fc Block™) | 2.4G2 | BD | 553142 |
|  | Anti-mouse CD11b APC | ICRF44 | BioLegend | 301350 |
| SIRPα genotyping assay | PBMCs from healthy donors |  | AllCells, LLC |  |
|  | 3730 Genetic Analyzer |  | ThermoFisher |  |
|  | SnapGene Viewer |  | SnapGene by Dotmatics |  |

Abbreviations: CFSE, carboxyfluorescein succinimidyl ester; FACS, fluorescence-activated cell sorter; M-CSF, macrophage colony-stimulating factor; PBMC, peripheral blood mononuclear cell; RO, receptor occupancy; SIRPα, signal regulatory protein alpha; SPR, surface plasmon resonance.

**SUPPLEMENTAL TABLE 2** Primers used for SIRPα genotyping using Sanger sequencing

| 1 | 5’-ACACAGAGGATCACGTAAGGATGA |
| --- | --- |
| 2 | 5’-GAGGGTCCAGGCATTCAAAC |
| 3 | 5’-GACAAGTCCGTATCA |
| 4 | 5’-CTTAAACTCCGTGTCAG |
| 5 | 5’-AAACCAGAGGCAAAGGAGGC |
| 6 | 5’-AAACCAGAGGCAAAGGAGGC |

Abbreviations: SIRPα, signal regulatory protein alpha.

**SUPPLEMENTAL TABLE 3** Grade 3/4 treatment-emergent laboratory abnormalities

| **n (%)** | **GS-0189**  **10 mg**  **(N = 1)** | **GS-0189**  **30 mg**  **(N = 1)** | **GS-0189**  **100 mg**  **(N = 4)** | **MDE**  **Total**  **(N = 6)** | **GS-0189**  **100 mg + rituximab**  **(N = 3)** |
| --- | --- | --- | --- | --- | --- |
| Any grade 3/4 abnormality | 0 | 0 | 1 (25.0)^a^ | 1 (16.7) | 3 (100.0) |
| Haemoglobin decreased | 0 | 0 | 1 (25.0) | 1 (16.7) | 0 |
| Leukocytes decreased | 0 | 0 | 1 (25.0) | 1 (16.7) | 0 |
| Lymphocytes decreased | 0 | 0 | 1 (25.0) | 1 (16.7) | 3 (100.0)^b^ |
| Neutrophils decreased | 0 | 0 | 1 (25.0) | 1 (16.7) | 0 |

^a^ All four of the laboratory abnormalities below occurred in a single patient in cycle 1. All were grade 3, and all had returned to baseline levels or below by cycle 1 day 8.

^b^ Lymphocyte decreases were transient.

Abbreviations: MDE, monotherapy dose escalation.

*Note:* Treatment-emergent lab abnormalities were values that increased at least 1 toxicity grade from baseline at any post-baseline time point, up to 30 days after permanent discontinuation of study drug. If the baseline value was missing, any abnormality of at least grade 1 within the time frame was considered as treatment emergent.

**SUPPLEMENTAL FIGURE 1** Study Schema. Patients were enrolled first in the monotherapy dose escalation (MDE) cohort using a modified accelerated dose titration design for the first 2 dose cohorts, followed by a standard 3+3 dose escalation design for all subsequent dose cohorts. The first patient was planned to be enrolled at the 10 mg dose level; if that patient did not have a Grade 2 or dose-limiting toxicity (DLT) during the first course of treatment, the next patient would be enrolled at the 30 mg dose level. If a grade 2 toxicity or DLT is reported, 2 more patients would be enrolled at the same dose level and subsequent enrollment will occur using a 3+3 design. According to the 3+3 design, if 1 of 3 patients has DLT(s) in any dose cohort, the cohort will expand to up to 6 patients. If ≥ 2 of the 6 patients have DLT(s), the acceptable DLT rate will have been exceeded, and patients at a lower dose cohort may be evaluated. The MTD level will be defined as the highest dose level tested with a DLT rate of less than 33% in at least 6 patients. Enrollment will proceed to the combination dose escalation (CDE) cohorts at the MTD of the MDE cohorts or 100 mg GS-0189 if no MTD is observed.


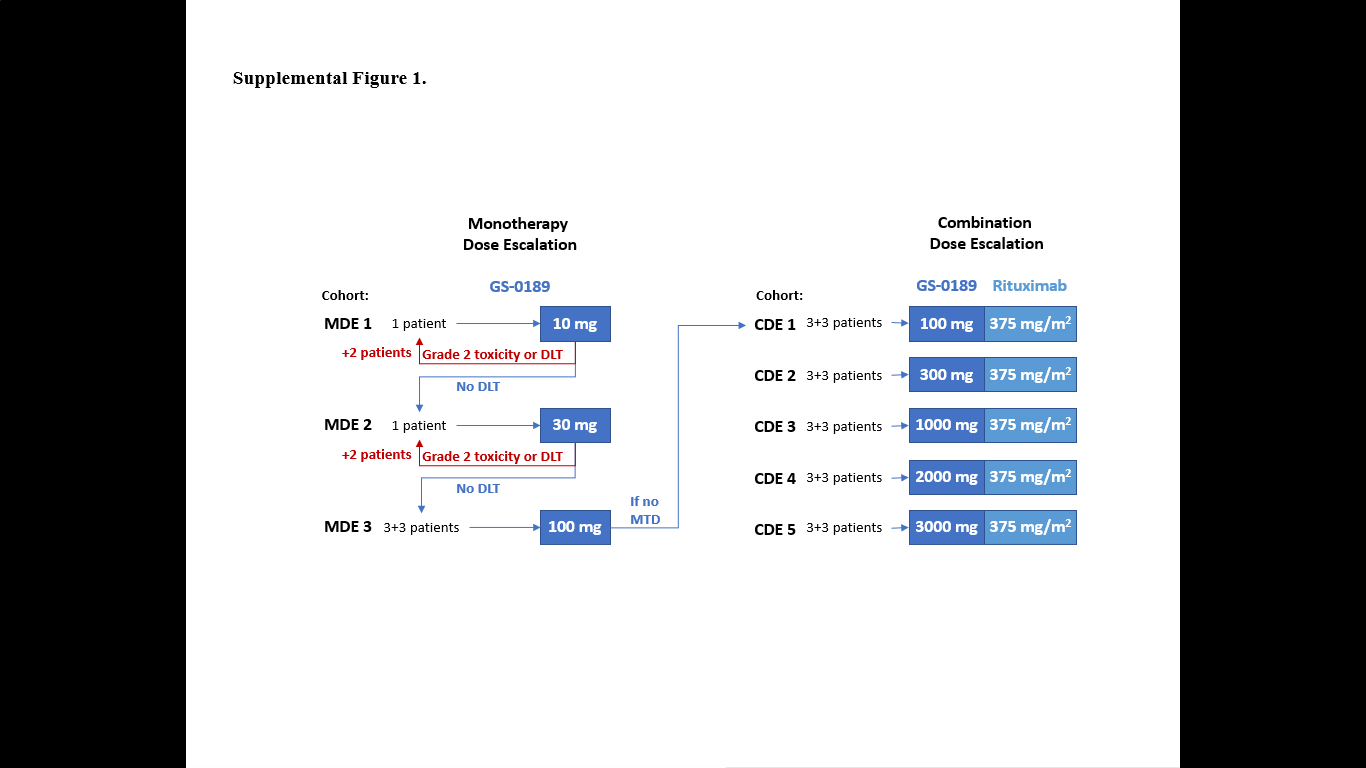


**SUPPLEMENTAL FIGURE 2** Receptor occupancy results from CDE1 cohort during treatment. Receptor saturation was observed 1-hour post-infusion of GS-0189 in 2 subjects, but significant reduction of GS-0189 binding was observed at days 8 and 15 post-infusion.
Abbreviations: CD, cluster of differentiation; CDE, combination dose escalation; RO, receptor occupancy


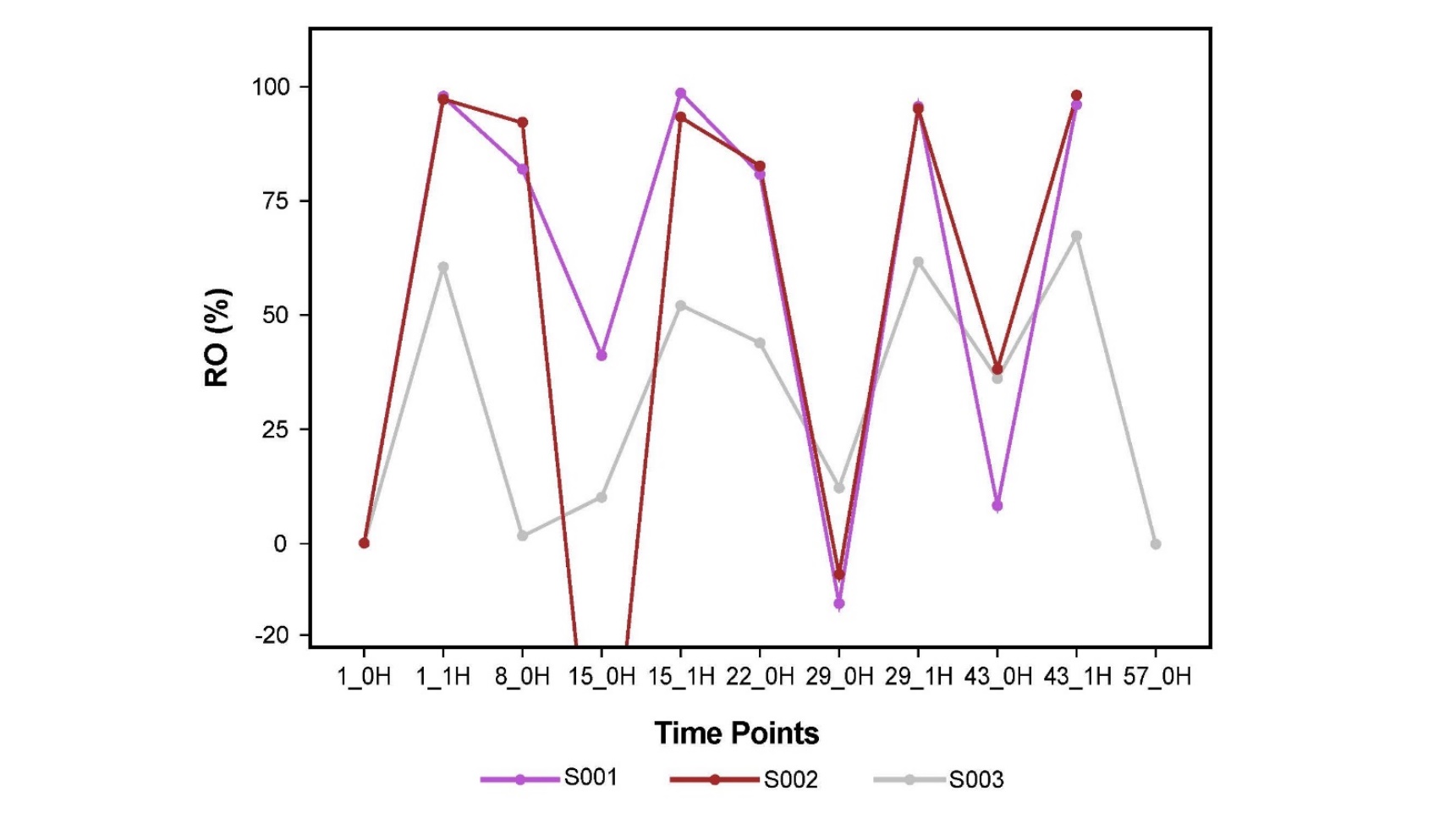


**SUPPLEMENTAL FIGURE 3** Phagocytosis induced by anti-SIRP and anti-CD20 antibodies. CFSE-labeled Raji cells were co-cultured with macrophages isolated from healthy donor PBMCs that were homozygous for SIRPα^V1^ or SIRPα^V2^ as described in the legend. Treatment with doses of GS-0189 as indicated in the legend induced phagocytosis in combination with 0.1 µg/mL of rituximab.
Abbreviations: CD, cluster of differentiation; CFSE, carboxyfluorescein succinimidyl ester; PBMC, peripheral blood mononuclear cell; SIRP, signal regulatory protein; SIRPα, SIRP alpha; v, variant


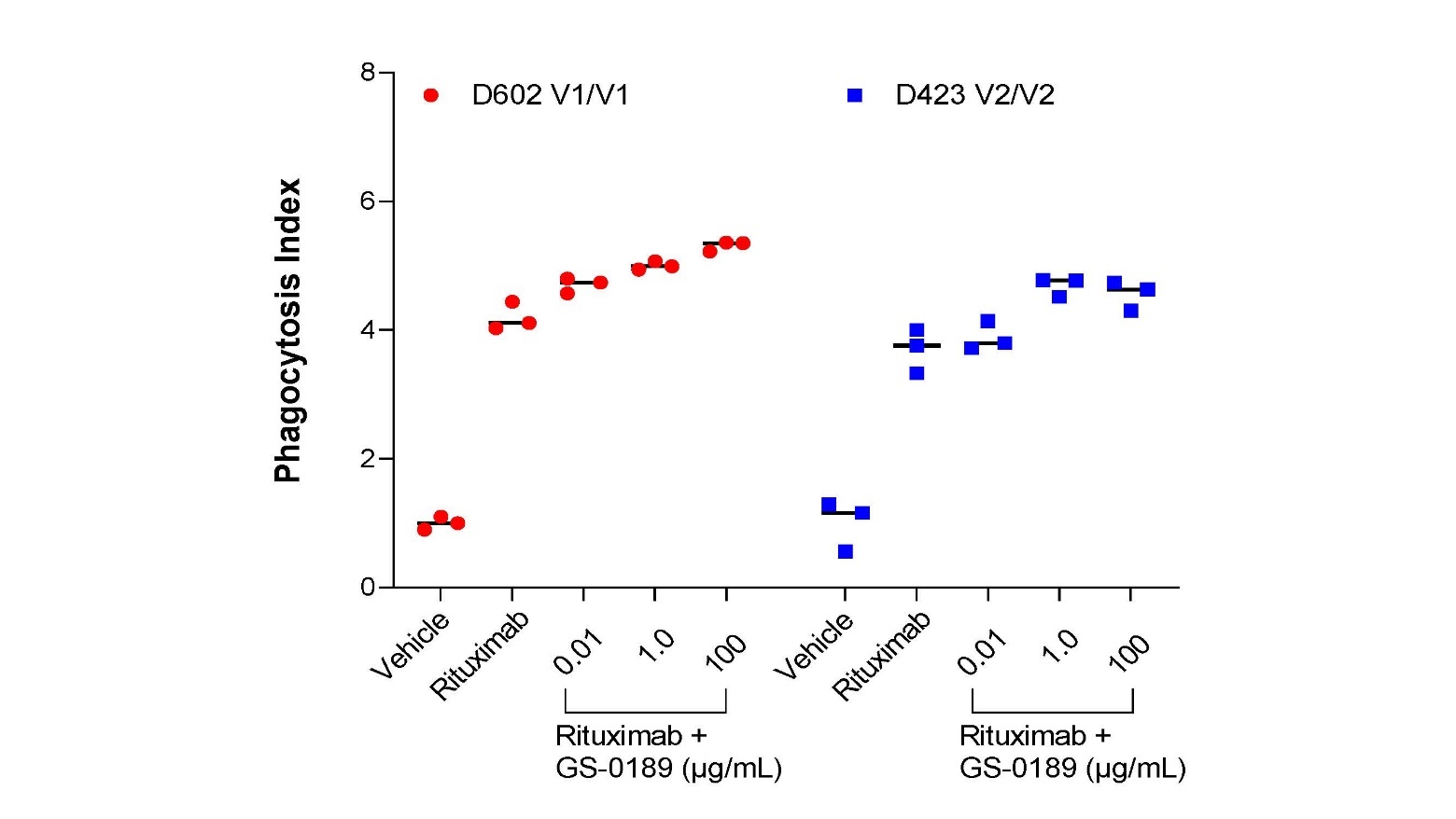


**SUPPLEMENTAL FIGURE 4** Phagocytosis of DLD-1 cells are dose-dependently induced by KWAR23 with similar potencies across donors**.** CFSE-labeled DLD-1 cells were co-cultured for 2 hours with macrophages isolated from healthy donor PBMCs that were homozygous or heterozygous for SIRPα^V1^ or SIRPα^V2^. KWAR23 mAb dose-dependently increased phagocytosis, with doses 10 ug/mL and above affording maximum phagocytic capacity. The frequency of CD11b^+^ macrophages positive for CFSE was normalised to vehicle to generate the phagocytic index. Samples were tested in duplicate and presented as mean ± SD.
Abbreviations: CD, cluster of differentiation; CFSE, carboxyfluorescein succinimidyl ester; mAb, monoclonal antibody; PBMC, peripheral blood mononuclear cell; SD, standard deviation; SIRPα, signal regulatory protein alpha; v, variant


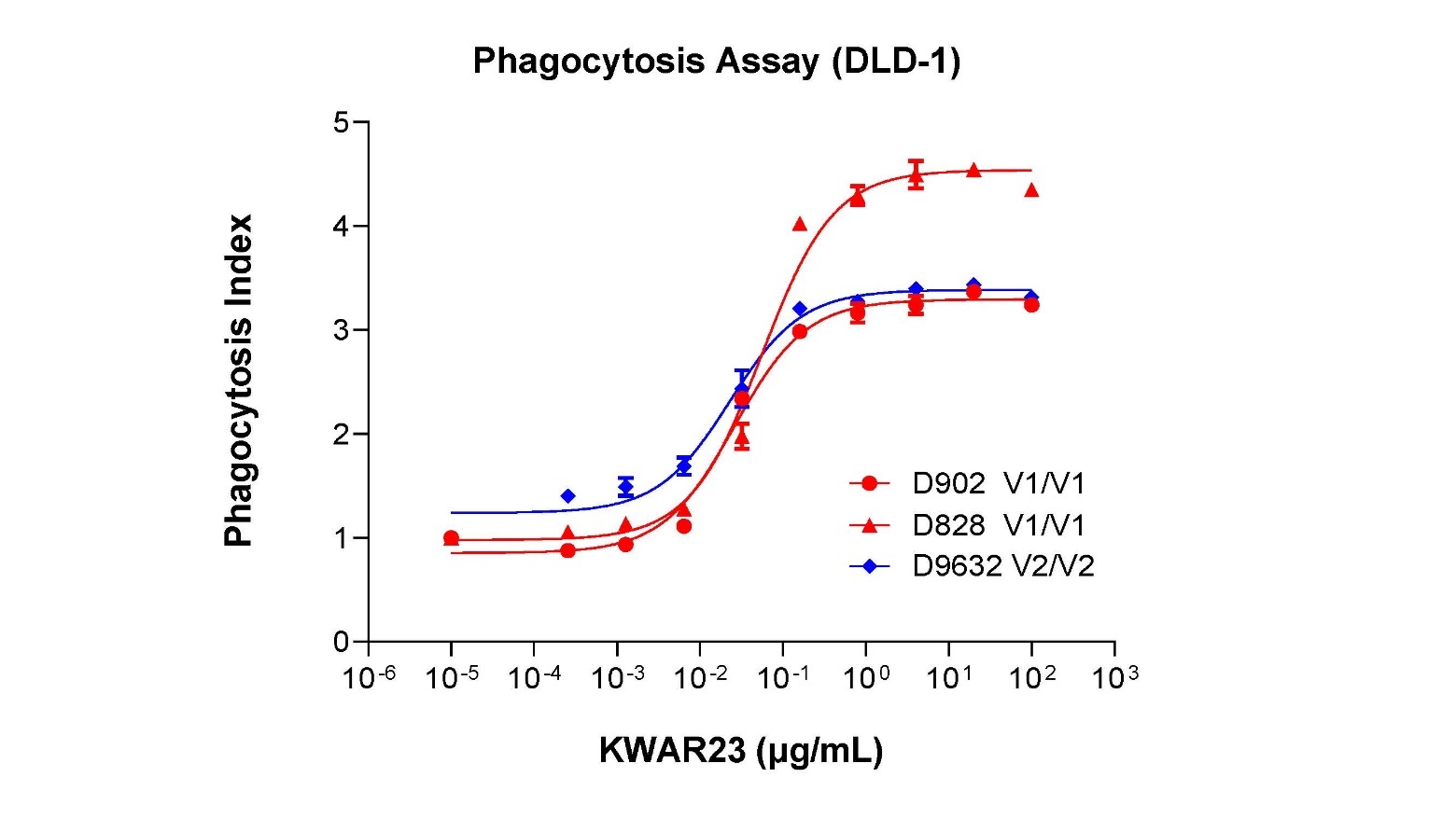

Supplement: Supplementary file 1 — Supporting Information [file JHA2-4-370-s001.docx]
